# Metronomic chemotherapy with daily, oral etoposide plus bevacizumab for recurrent malignant glioma: a phase II study

**DOI:** 10.1038/sj.bjc.6605412

**Published:** 2009-11-17

**Authors:** D A Reardon, A Desjardins, J J Vredenburgh, S Gururangan, J H Sampson, S Sathornsumetee, R E McLendon, J E Herndon, J E Marcello, J Norfleet, A H Friedman, D D Bigner, H S Friedman

**Affiliations:** 1 Department of Surgery, The Preston Robert Tisch Brain Tumor Center, Duke University Medical Center, Durham, NC 27710, USA; 2 Department of Pediatrics, The Preston Robert Tisch Brain Tumor Center, Duke University Medical Center, Durham, NC 27710, USA; 3 Department of Medicine, The Preston Robert Tisch Brain Tumor Center, Duke University Medical Center, Durham, NC 27710, USA; 4 Department of Pathology, The Preston Robert Tisch Brain Tumor Center, Duke University Medical Center, Durham, NC 27710, USA; 5 Cancer Center Biostatistics, The Preston Robert Tisch Brain Tumor Center, Duke University Medical Center, Durham, NC 27710, USA

**Keywords:** malignant glioma, glioblastoma, angiogenesis, bevacizumab, vascular endothelial growth factor, metronomic chemotherapy

## Abstract

**Background:**

We evaluated bevacizumab with metronomic etoposide among recurrent malignant glioma patients in a phase 2, open-label trial.

**Methods:**

A total of59 patients, including 27 with glioblastoma (GBM) and 32 with grade 3 malignant glioma, received 10 mg kg^−1^ bevacizumab biweekly and 50 mg m^−2^ etoposide daily for 21 consecutive days each month. The primary end point was a 6-month progression-free survival, and secondary end points included safety and overall survival. Vascular endothelial growth factor (VEGF), VEGFR-2, carbonic anhydrase 9 (CA9) and hypoxia-inducible factor-2*α* (HIF-2*α*) were assessed semiquantitatively in archival tumours using immunohistochemistry and were correlated with outcome.

**Results:**

Among grade 3 and GBM patients, the 6-month progression-free survivals were 40.6% and 44.4%, the radiographic response rates were 22% and 37% and the median survivals were 63.1 and 44.4 weeks, respectively. Hypertension predicted better outcome among both grade 3 and GBM patients, whereas high CA9 and low VEGF were associated with poorer progression-free survival (PFS) among those with GBM. The most common grade ⩾3 adverse events included neutropaenia (24%), thrombosis (12%), infection (8%) and hypertension (3%). Two patients had asymptomatic, grade 1 intracranial haemorrhage and one on-study death occurred because of pulmonary embolism.

**Conclusion:**

Bevacizumab with metronomic etoposide has increased toxicity compared with previous reports of bevacizumab monotherapy. Its anti-tumour activity is similar to that of bevacizumab monotherapy or bevacizumab plus irinotecan. (ClinicalTrials.gov: NCT00612430).

Malignant gliomas, including the most common subtype, grade 4 glioblastoma (GBM), as well as WHO grade 3 tumours such as anaplastic astrocytoma (AA), remain essentially fatal malignancies (Wen and Kesari, 2008). The median survival for GBM patients is <15 months after external beam radiotherapy and temozolomide after maximal safe resection (Stupp *et al*, 2005), whereas survival for most grade 3 patients is 2–3 years (See and Gilbert, 2004).

Profound angiogenesis, a hallmark of malignant glioma, results from a ‘perfect storm’ of hypoxia and hypoxia-independent aberrant activation of cell signalling pathways that markedly upregulate vascular endothelial growth factor (VEGF) expression (Jain *et al*, 2007). Anti-angiogenic strategies targeting VEGF are active among recurrent GBM patients. Daily administration of AZD2171 (cediranib, AstraZeneca Pharmaceuticals, London, UK), a potent oral, pan-VEGF receptor inhibitor that also inhibits platelet-derived growth factor (PDGF) receptor and c-Kit, achieved an overall radiographic response (ORR) of 56% and a median progression-free survival (PFS) of 3.96 months (Batchelor *et al*, 2007). Bevacizumab, a humanised monoclonal antibody against VEGF (Avastin, Genentech Pharmaceuticals, South San Francisco, CA, USA), was originally reported to achieve dramatic radiographic responses when combined with the topoisomerase-1 inhibitor irinotecan among recurrent GBM patients (Stark-Vance, 2005). In a formal phase II study of this regimen, Vredenburgh noted a 61% ORR, 46% 6-month PFS (6-PFS) and a median overall survival (OS) of 10 months among heavily pre-treated, recurrent GBM patients (Vredenburgh *et al*, 2007a, 2007b). The anti-tumour activity of bevacizumab with and without irinotecan was confirmed in two follow-up, prospective studies (Friedman *et al*, 2009; Kreisl *et al*, 2009) that recently led to accelerated approval of bevacizumab monotherapy for recurrent GBM by the US Food and Drug Administration. In addition, we have also recently noted that bevacizumab plus irinotecan is active for recurrent grade 3 malignant glioma patients (Desjardins *et al*, 2008). These studies, as well as additional retrospective series (Bokstein *et al*, 2008; Kang *et al*, 2008; Zuniga *et al*, 2009), have also confirmed the safety profile of this regimen for malignant glioma patients.

This study was conducted to evaluate the anti-tumour activity of etoposide, a topoisomerase-2 inhibitor, when administered on a protracted, oral, ‘metronomic’ dosing schedule, in combination with standard bevacizumab administration. Etoposide has modest activity among recurrent malignant glioma patients as either single-agent therapy or in combination with other chemotherapeutics (Chamberlain and Grafe, 1995; Fulton *et al*, 1996; Kesari *et al*, 2007). We hypothesised that etoposide administered on protracted, daily schedule may exert an effect as a metronomic chemotherapeutic (Kerbel and Kamen, 2004) and thereby enhance the anti-angiogenic and anti-tumour effect of bevacizumab in this patient population.

## Patients and methods

### Protocol objectives

Our primary objective was to estimate the 6-month PFS among adults with recurrent malignant glioma treated with protracted, oral etoposide plus bevacizumab. Secondary objectives included evaluation of the safety and tolerability of this regimen and evaluation of the radiographic response, PFS and OS associated with this regimen in this patient population.

### Patient eligibility

Patients were required to have histological confirmation of grade 3 or 4 malignant glioma that was recurrent after previous radiation or chemotherapy. Patients with previous low-grade glioma were eligible if histological transformation to malignant glioma was confirmed. Eligible patients were also at least 18 years of age, had a Karnofsky performance status (KPS) ⩾60% and were on a stable corticosteroid dose for at least 1 week. Additional enrollment criteria included: haematocrit >29%; absolute neutrophil count >1500 cells *μ*l^−1^; platelet count >100 000 cells *μ*l^−1^; and serum creatinine <1.5 mg per 100 ml, aspartate aminotransferase and bilirubin <1.5 times the institutional upper limit of normal. At least 4 weeks between surgical resection or chemotherapy, and at least 12 weeks between radiotherapy, and enrollment were required. All patients provided informed consent.

Patients were excluded for: >3 recurrences; uncontrolled hypertension; therapeutic anticoagulation use; acute haemorrhage on baseline MRI; urine protein:creatinine ratio >1; pregnancy or nursing; previous bevacizumab or etoposide; active infection requiring intravenous antibiotics; and previous stereotactic radiosurgery, radiation implants or radiolabelled monoclonal antibody therapy, unless there was unequivocal disease progression (such as a new lesion or biopsy-proven recurrence).

### Treatment design

Patients were stratified by histopathology into those with GBM and those with grade 3 malignant glioma. All patients were administered 50 mg m^−2^ of etoposide daily for 21 consecutive days of each 28-day cycle. Bevacizumab was administered at 10 mg kg^−1^ intravenously every 14 days. Study therapy was planned to discontinue after 12 cycles. However, patients were given the option to continue study therapy for 6 additional months if they were felt to potentially derive further therapeutic benefit. Specifically, such patients included those who had ongoing radiographic or clinical improvement after 12 months or those who had persistent contrast enhancement that was accompanied by hypermetabolic activity on [^18^F]fluorodeoxyglucose positron emission tomography (^18^FDG-PET) imaging. Study therapy was discontinued for unacceptable toxicity, tumour progression, study non-compliance or consent withdrawal.

### Response evaluation

Study investigators determined response by neurological examination and contrast-enhanced MRI previous to every other treatment cycle. A complete response (CR) required disappearance of all enhancing tumours with corticosteroid discontinuation and neurological stability or improvement. A partial response (PR) required ⩾50% reduction in size (product of largest perpendicular diameters) of enhancing tumour with stability or improvement of neurological status and corticosteroids. Complete and partial responses also required stable or improved signal abnormality on fluid-attenuated inversion recovery (FLAIR) sequences and confirmation on consecutive scans at least 4 weeks apart. Progressive disease (PD) was defined by any of the following: ⩾25% increase on enhancing tumour; a new enhancing lesion; or clear worsening of FLAIR signal abnormality or significant clinical decline that was not attributable to co-morbid event or concurrent medication. Stable disease was defined as any assessment not meeting CR, PR or PD criteria. The inclusion of FLAIR signal changes in the classification of response in this study was based on the growing recognition that some malignant glioma patients undergoing therapy with VEGF or VEGFR inhibitors may have significant improvement in contrast enhancement, yet have extensive progressive changes on T2 or FLAIR sequences, often accompanied by marked neurological decline, which is felt to be indicative of progressive infiltrative tumour (Bokstein *et al*, 2008; Norden *et al*, 2008; van den Bent *et al*, 2009).

### Dose modification and retreatment criteria

Daily etoposide dose was reduced by 25 mg for grade 3 thrombocytopenia, grade 4 neutropaenia or other related grade ⩾3 non-haematologic events. Bevacizumab was discontinued for uncontrollable hypertension, grade 2 or greater haemorrhage, arterial thrombosis, wound dehiscence requiring surgical intervention, intestinal perforation, grade 4 venous thrombosis, proteinuria or congestive heart failure. Bevacizumab was held until other grade 3, related events resolved to a grade ⩽ 1.

Initiation of each cycle required an absolute neutrophil count (ANC) ⩾1000 mm^−3^; a platelet count ⩾100 000 mm^−3^; aspartate aminotransferase (AST), bilirubin and creatinine less than twice the institutional upper limit of normal; and resolution of any related grade ⩾3 event to a grade ⩽1.

### Tumour marker analysis

Immunohistochemistry staining of archival, paraffin-embedded tumour sections was performed and analysed for VEGF, VEGFR-2/KDR as well as two markers of hypoxia, hypoxia-inducible factor 2*α* (HIF-2*α*) and carbonic anhydrase 9 (CA9), as previously described (Sathornsumetee *et al*, 2008) by a neuropathologist who was unaware of clinical and imaging outcome. A semiquantitative score was derived from an intensity score of the reactivity product (absent, 0; mild, 1; moderate, 2; strong, 3) related with endogenous positive controls multiplied by distribution score (percentage of reactive cells in tumour).

### Statistical considerations

The primary aim of this study was to evaluate the 6-PFS rate of bevacizumab plus protracted, oral etoposide among recurrent malignant glioma patients. Yung *et al* (2000) reported a 6-PFS rate of 21% (95% CI 13–29) among recurrent GBM patients treated with temozolomide at first recurrence. Given that the prognosis of our patient population was expected to be poorer than that reported by Yung *et al*, the sample size goal of 27 GBM patients for this study was chosen to provide 82% power to differentiate between 6-PFS of 5 and 20% with a type I error of 0.04. Similarly, the 6-PFS of temozolomide for recurrent grade 3 malignant glioma patients as reported by Yung *et al* is 46% (95% CI 38–54). Given that the prognosis of our grade 3 patients was again expected to be poorer than that reported, a sample size goal of 32 recurrent grade 3 patients was chosen to provide 80% power to differentiate between a 6-PFS of 20 and 40% with a type I error rate of 0.04. The benchmark set by temozolomide was chosen as the historical comparator for our study rather than the outcome reported on previous bevacizumab studies because the latter had not been validated in a multi-institutional setting when this study was designed.

‘Stopping rules’ for poor efficacy and unacceptable toxicity were incorporated for each stratum. Specifically, if ⩾10 of the first 16 patients per stratum progressed or died within 2 months of study initiation, further accrual would be suspended. In addition, if 6 or more of the first 16 patients per stratum experienced unacceptable toxicity, defined as grade ⩾4 non-haematological events, further accrual would be suspended.

Progression-free survival and OS were measured from the cycle 1 start date and summarised using Kaplan–Meier estimator including 95% CIs. For each cohort, PFS distribution was compared between the following subgroups using the log-rank test: patients <50 years old *vs* those ⩾50 years; patients with a KPS <90 *vs* those with a KPS ⩾90; patients with >1 previous episode of progression *vs* those with 1 previous progression; and patients who received >1 previous chemotherapeutic *vs* those who received only 1 previous chemotherapeutic. We also sought to determine whether hypertension was associated with outcome. For these purposes, hypertension was defined as sustained grade 1 for at least 4 weeks, grade ⩾2 or the initiation or increase in anti-hypertensive medications. Log-rank tests were conducted comparing patients who developed hypertension with those who did not relative to OS and PFS.

The effect of each tumour marker on overall and PFS was evaluated using separate Cox's proportional hazard models. Hazard ratios and the *P*-value from Wald's chi-square tests were determined for each model. The effect of each tumour marker (CA9, VEGF, VEGFR-2 and HIF2*α*) on PFS-6 and radiographic response was evaluated using logistic regression models. Odds ratios and the *P*-value from Wald's chi-square tests were determined for each model. The effect of each categorical marker on PFS-6 and radiographic response was also estimated using Fisher's exact test.

## Results

### Patient characteristics

A total of 59 patients were enrolled between April 2007 and January 2008, including 32 with grade 3 malignant glioma and 27 with GBM (Table 1).

Most patients had bulky disease at enrollment, given that only 7% underwent a gross total resection of enhancing disease before enrollment. Patients who underwent a GTR were not evaluable for radiographic response. In general, patients were significantly pretreated with 53% enrolling at either second or third progression, whereas 73% had received two or more previous chemotherapeutic agents.

As of 15 May 2009, four GBM patients (15%) remain alive, including three with progressive disease and one with stable disease in the tenth month of the study therapy. Again, 16 grade 3 malignant glioma patients (50%) remain alive, including 2 who continue on-study therapy, 5 who are off study without recurrence and 9 with progressive disease. In total, 39 patients (66%) have died. Median follow-up was 91.2 weeks for GBM patients, and 69.9 weeks for grade 3 malignant glioma patients.

### Study drug administration and safety

Study drug administration and compliance with treatment for the intent-to-treat (ITT) study population were excellent. A total of 389 cycles of therapy were administered, including a median of 6.6 (range 1–18) cycles for the grade 3 malignant glioma patients and 6.6 (range 1–20) cycles for the GBM patients, respectively.

All patients were assessable for toxicity. As the adverse event profiles were similar for both patient cohorts, a pooled summary of toxicity is provided in Table 2. Significant haematological toxicity included neutropaenia (grade 3, *n*=9 (15%); grade 4, *n*=5 (8%)), thrombocytopaenia (grade 3, *n*=2 (3%)) and anaemia (grade 3, *n*=1 (2%)). Most non-haematologic toxicities were of grade 2. The most common serious non-haematologic events were thrombosis (grade 3, *n*=4 (7%); grade 4, *n*=2 (3%); grade 5, *n*=1 (2%)), infection (grade 3, *n*=4 (7%); grade 4, *n*=1 (2%)) and hypertension (grade 3, *n*=1 (2%); grade 4, *n*=1 (2%)). Haemorrhage was limited to two patients with grade 1 central nervous system (CNS) bleeds detected on surveillance MRI, and one patient with grade 2 GI bleeding from haemorrhoids. A single on-study death, attributed to pulmonary embolism, occurred.

A total of nine grade 3 malignant glioma patients (28%) required etoposide dose modification, including eight patients for grade 3 or 4 neutropaenia and one patient with grade 2 peripheral neuropathy. Two GBM patients (7%) required etoposide dose modification including one for grade 4 neutropaenia and one for peripheral neuropathy. Four patients (13%) on the grade 3 arm discontinued therapy because of toxicity including one patient with persistent grade 3 proteinuria after 14 cycles of therapy, 1 patient with dehiscence of a previously healed craniotomy incision after 7 cycles of therapy and the two patients described above with grade 1 CNS haemorrhage. Three GBM patients (11%) discontinued therapy because of adverse events, all of whom had pulmonary emboli.

### Outcome

**Figure 1:**
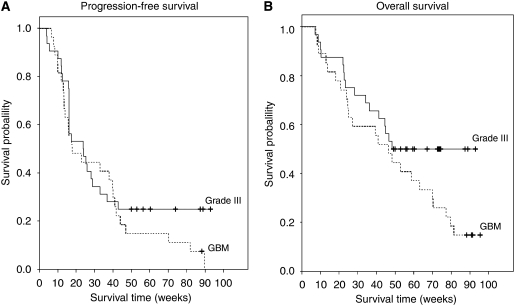
Kaplan–Meier plots of progression-free survival (**A**) and overall survival (**B**) for grade 3 malignant glioma and glioblastoma patients.

**Figure 2:**
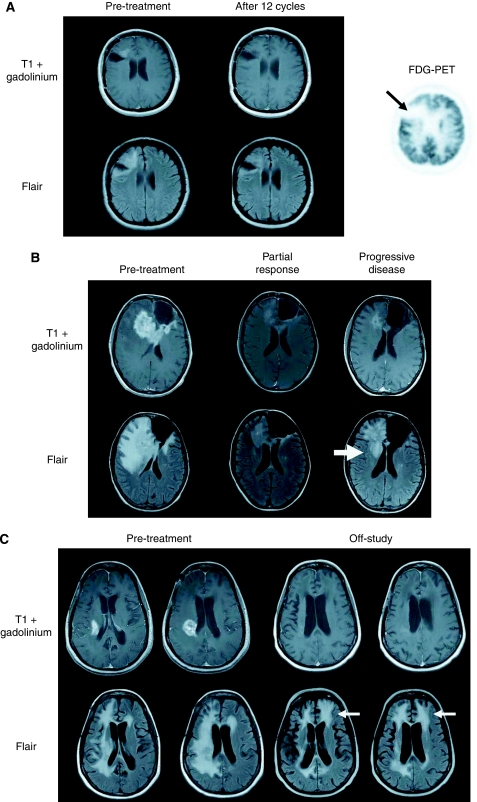
Baseline and post-treatment magnetic resonance imaging of a representative, responding glioblastoma patient including post-contrast axial T1-weighted and fluid-attenuated inversion recovery (FLAIR) images. [^18^F]fluorodeoxyglucose positron-emission tomography imaging at the completion of 12 cycles of therapy shows no evidence of hypermetabolic activity (arrow). (**A**) Baseline and off-study magnetic resonance imaging showing worsened FLAIR signal abnormality, indicating an infiltrative, microscopic pattern of treatment failure (arrows) involving the isolateral hemisphere (**B**) and the contralateral hemisphere (**C**), despite improved contrast enhancement.

The median OS, PFS, 6-PFS rate and the rate of radiographic response are summarised in Table 3 and Figure 1. Outcome comparisons were not performed for subsets of grade 3 malignant glioma patients because of the small sample size. Complete and partial responses were observed in one (4%) and five (19%) GBM patients, and two (7%) and five (17%) grade 3 patients. Stable disease was the best response in 19 (73%) and 21 (72%) of GBM and grade 3 patients, respectively, whereas only two GBM (7%) and four grade 3 (13%) patients progressed at first evaluation. In total, 10 patients (17%) completed 12 cycles of therapy and eight of these patients (80%) had no evidence of hypermetabolic activity on [^18^F]FDG-PET imaging (Figure 2A). Among six patients (10%) who completed 1 year of planned therapy, five remain alive, including four without tumour recurrence (all grade 3 malignant glioma) and one (GBM) with recurrent tumour; one GBM patient who completed a year of therapy subsequently died of progressive tumour.

Although limited by sample size, the development of grade ⩾1 hypertension was linked with improved outcome. Among GBM patients who developed hypertension, median OS was not reached and 1-year OS was 100%, whereas median and 1-year OS were 39.4 weeks and 34.8%, respectively, for those who did not develop hypertension (*P*=0.0095). A trend towards improved OS was also noted among grade 3 patients who developed hypertension and all of these patients also remain alive. In addition, PFS was longer for grade 3 patients who developed hyptertension (*P*=0.024). Among GBM patients, median and 6-PFS were 61 weeks and 100%, respectively, for those who developed hypertension when compared with 16 weeks and 34.8% for those who did not; however, these differences did not achieve statistical significance (*P*=0.123).

None of the demographic or pre-treatment characteristics had an association with PFS within either patient cohort, although a trend towards improved PFS was noted among grade 3 patients with a KPS ⩾90 (*P*=0.054). In all, eight patients developed hypertension (four GBM and four grade 3 MG, Table 2). A trend towards improved survival was observed in these patients, but the sample size was too small for subgroup analysis.

The 27 patients who achieved stable disease or radiographic response were on corticosteroids at study initiation, and 10of them (37%) decreased corticosteroids while on study, including five patients (19%) who successfully tapered off corticosteroids completely.

### Tumour markers

In all, 12 GBM patients (44%) and 11 grade 3 malignant glioma patients (34%) had adequate tumour material for marker expression analysis (Supplementary Table 1, Supplementary Figure 1). All tumours expressed VEGF and VEGFR2 but the range of expression varied from 10 to 80% for VEGF and 2 to 80% for VEGFR2. Carbonic anhydrase 9 was detected in 11 of 12 (92%) GBM tumours (percentage of positive cells: 1–80%) and in 8 of 11 (73%) grade 3 tumours (range of positive cells: 1–40%). Hypoxia-inducible factor-2*α* was detected in 8 of 8 GBM tumours (range of positive cells: 0.01–4%) and in 8 of 10 grade 3 tumours (range of positive cells: 0.1–20%). All markers were more commonly expressed by GBM tumours compared with grade 3 tumours.

Low CA9 expression (⩽10% of cells; *P*=0.04) and increased VEGF expression (>30% of cells; *P*=0.006) were associated with better PFS among GBM patients (Supplementary Table 1). None of the tumour markers were predictive of outcome among grade 3 tumours.

### Pattern of failure

A total of 22 grade 3 malignant glioma patients (69%) and 21 GBM patients (78%) developed radiographic evidence of progressive disease. The pattern of recurrence included worsened enhancement plus FLAIR changes at the primary tumour site in 15 (68%) of the grade 3 patients and 13 (62%) GBM patients. Clinical decline and progressive local FLAIR changes without worsened enhancement were noted in four grade 3 patients (18%) and six GBM patients (29%), respectively (Figures 2B and C). In addition, three grade 3 patients (14%) and two GBM patients (10%) had new distant sites of disease (defined as >2 cm away from previous enhancement without contiguous FLAIR signal abnormality) at progression.

## Discussion

We report the first clinical trial evaluating metronomic chemotherapy combined with bevacizumab for recurrent malignant glioma patients. We hypothesised that etoposide, administered on a metronomic or protracted, daily dosing schedule would enhance the anti-tumour activity of bevacizumab based on potentially complimentary mechanisms of anti-angiogenic action. Bevacizumab binds VEGF-A, preventing activation of VEGFR1 and VEGFR2, whereas metronomic chemotherapy may exert an effect through a number of anti-angiogenic actions, including induction of endogenous angiogenesis inhibitors such as thrombospondin-1 (Damber *et al*, 2006), enhancement of endothelial cells apoptosis (Bocci *et al*, 2002) and decreasing the mobilisation and viability of circulating endothelial progenitor cells (Bertolini *et al*, 2003). Preclinical studies confirm that metronomic etoposide is preferentially cytotoxic against tumour endothelial cells compared with tumour cells *in vitro* (Drevs *et al*, 2004), and that metronomic etoposide plus anti-angiogenic therapy prolongs survival in orthotopic, intracranial U87 GBM xenografts compared with conventionally dosed chemotherapy with or without anti-angiogenic therapy (Bello *et al*, 2001). Clinically, several studies using metronomic dosing of etoposide have shown evidence of modest activity among recurrent malignant glioma patients (Chamberlain and Grafe, 1995; Fulton *et al*, 1996; Kesari *et al*, 2007), as well as other cancer patient populations (Correale *et al*, 2006; Twardowski *et al*, 2008). To date, the only published studies evaluating metronomic chemotherapy plus bevacizumab have involved patients with recurrent breast and ovarian cancer, and show anticancer benefit (Dellapasqua *et al*, 2008; Garcia *et al*, 2008; Garcia-Saenz *et al*, 2008).

Our study revealed that metronomic etoposide plus bevacizumab has encouraging outcome when compared with established benchmarks. Specifically, for recurrent GBM patients, our 6-PFS rate and median OS were higher than those reported with temozolomide at first recurrence (Yung *et al*, 2000), as well as several studies with etoposide (Fulton *et al*, 1996; Chamberlain and Kormanik, 1999) and historical series of salvage regimens.(Wong *et al*, 1999; Ballman *et al*, 2007; Lamborn *et al*, 2008). In addition, the outcomes of our study did not differ significantly to that achieved with bevacizumab plus irinotecan in a single-institution, phase 2 study (Vredenburgh *et al*, 2007b). It is noteworthy that patients in that study as well as in this study were heavily pretreated with a median of two previous episodes of progressive disease.

A major remaining question is whether chemotherapy, including metronomic, adds benefit over bevacizumab alone for recurrent malignant glioma patients. Although a recently reported phase II study randomised GBM patients at either first or second recurrence to bevacizumab monotherapy or bevacizumab plus irinotecan, this study was not powered to compare treatment arms and randomisation served solely to eliminate treatment assignment bias. The rate of radiographic response by blinded centralised review and 6-PFS were 38 and 50% for patients treated with combination therapy and 28 and 43% for those treated with bevacizumab monotherapy. Although median OS did not differ between the arms, monotherapy patients were also allowed to crossover to the combination arm at progression and continue study therapy. Of concern, the overall toxicity appeared increased in the combination cohort (Friedman *et al*, 2009). A recent single-arm study confirmed the anti-tumour efficacy of bevacizumab monotherapy among recurrent GBM patients (Kreisl *et al*, 2009). The results of the current study are comparable with those reported previously for bevacizumab-based therapy (Table 3). Furthermore, the addition of chemotherapy to bevacizumab, as shown in previous studies with irinotecan and in this study with metronomic etoposide, can lead to increased toxicity.

In this study we also noted that bevacizumab plus metronomic etoposide is active among recurrent grade 3 malignant glioma patients. It is noteworthy that there are no data with bevacizumab monotherapy for this subset of malignant glioma patients, but our study results compare favourably with those achieved with bevacizumab plus irinotecan (Desjardins *et al*, 2008) (Table 3), and are superior to those reported in two meta-analyses of salvage regimens (Wong *et al*, 1999; Lamborn *et al*, 2008).

As noted in other clinical trials (Chamberlain and Grafe, 1995; Fulton *et al*, 1996; Kesari *et al*, 2007), protracted daily etoposide was well tolerated and did not increase bevacizumab-specific toxicity. Grade 3 or 4 neutropaenia, the most common serious toxicity, occurred in approximately 24% of patients, but responded to dose modification. Grade 3 or 4 anaemia and thrombocytopaenia occurred rarely. The spectrum of non-haematological toxicity was comparable with that previously reported for bevacizumab in this patient population (Friedman *et al*, 2009) and appeared more favourable than that reported for bevacizumab plus irinotecan. Specifically, 7 patients (11%) in the current study discontinued therapy because of toxicity, whereas 11 patients (31%) in our single-institutional study (Vredenburgh *et al*, 2007b) and 14 patients (18%) in the randomised study (Friedman *et al*, 2009) discontinued bevacizumab plus irinotecan because of toxicity.

Corticosteroids, which are routinely administered to brain tumour patients to control symptoms due to cerebral oedema, cause a multitude of adverse sequellae that diminish quality of life, including marked weight gain, muscle loss, osteoporosis, hypertension, dysregulated glucose metabolism and immunosuppression (Wen *et al*, 2006). In this study, 37% of patients who were on chronic corticosteroid administration at the time of enrollment were able to taper their daily corticosteroid dose, including 19% who were able to completely discontinue corticosteroids. A similar ability to decrease chronic corticosteroid requirements has been noted in other studies evaluating anti-VEGF/R therapeutics (Batchelor *et al*, 2007; Vredenburgh *et al*, 2007a, 2007b; Desjardins *et al*, 2008).

Hypertension, a known complication of VEGFR signalling inhibition, is likely related with several factors, including diminished nitric oxide and prostacyclin synthesis (Scotland *et al*, 2005), diminished endothelial baroreceptor response (Yang *et al*, 2002), small artery and arteriole rarefaction (Ciuffetti *et al*, 2003) as well as increased vascular stiffness (Veronese *et al*, 2006). To our knowledge, this is the first clinical trial among malignant glioma patients to note an association between hypertension after VEGF-targeted therapy and improved outcome. However, this association should be interpreted cautiously and may have been because of a statistical coincidence, given the small number of patients who developed hypertension on this study. Clearly, our results require further validation in subsequent clinical trials. Nonetheless, hypertension has been associated with better outcome among other cancer patient populations after bevacizumab (Rixe *et al*, 2007; Schneider *et al*, 2008; Scartozzi *et al*, 2009), leading some to postulate that hypertension may serve as a biomarker of response (Jubb *et al*, 2006; van Heeckeren *et al*, 2007). Alternatively, it is possible that the development of hypertension may simply reflect longer bevacizumab exposure. We evaluated this possibility and noted that the interval from study initiation to hypertension was 6.4 weeks for GBM patients and 32 weeks for the grade 3 patients. The fact that both of these values were considerably less than the median PFS observed for each study cohort suggests that hypertension may be a predictive marker rather than simply reflecting prolonged bevacizumab exposure. We further analysed this association by reviewing our previous bevacizumab plus irinotecan trial for recurrent GBM (Vredenburgh *et al*, 2007b). In this series, similar to the current study, there was a trend towards improved median PFS, 6-PFS and OS among patients who developed hypertension compared with those who did not, but this analysis was also limited by small sample size. Nonetheless, further evaluation of hypertension as a potential predictor of bevacizumab activity among recurrent malignant glioma patients should be considered.

Our evaluation of tumour marker expression and outcome were clearly limited by the small number of analysed tumours as well as the use of archival tumour samples. Nonetheless, we noted that improved PFS was associated with lower CA9 expression as a marker of tumour hypoxia. It is noteworthy that we previously noted a similar association among recurrent malignant glioma patients treated with bevacizumab and irinotecan (Sathornsumetee *et al*, 2008). Of interest, we also noted that patients who had lower levels of VEGF, the target of bevacizumab, VEGF, seemed to respond less well with a lower PFS. Further analysis to validate these findings should be considered.

Our group and others have noted that potent VEGF inhibitors can decrease the permeability and intravenous contrast enhancement within 24–48 h (Batchelor *et al*, 2007; Desjardins *et al*, 2007). Although radiographic response among malignant glioma patients is traditionally based on changes in the largest bi-dimensional product of enhancement after gadolinium administration (Macdonald *et al*, 1990), anti-VEGF therapy can significantly and progressively worsen non-enhancing oedema on MRI that may be due to infiltrative disease, despite stable or even improved contrast-enhanced images (Ananthnarayan *et al*, 2008; Kang *et al*, 2008; Norden *et al*, 2008; Zuniga *et al*, 2009). We observed this pattern of progression in 17% of patients in the current study. Selection for a more invasive phenotype is supported by preclinical studies that revealed increased ‘satellites’ of infiltrative tumour that co-opt normal host vessels in orthotopic GBM xenografts treated with a VEGF monoclonal antibody (Rubenstein *et al*, 2000). Furthermore, as noted by others (Norden *et al*, 2008; Zuniga *et al*, 2009), we observed that new distant disease occurs in approximately 10% of patients. More diffuse, infiltrative, non-enhancing tumour at progression as well as the emergence of new, distant CNS sites of disease suggest that malignant gliomas may evolve resistance to VEGF-targeted therapeutics by adopting a more invasive phenotype that is relatively independent of VEGF signalling.

We performed the first study of bevacizumab administered with a metronomic chemotherapeutic (etoposide) and show that this regimen is safe and associated with encouraging clinical and survival benefit among recurrent GBM and grade 3 malignant glioma patients. Further studies evaluating this specific regimen as well as others, combining a metronomic chemotherapeutic plus a VEGF/R inhibitor, should be considered. Additional prospective studies to evaluate potential predictive biomarkers of anti-VEGF therapy for malignant glioma patients, including hypertension as well as tumour hypoxia and VEGF expression, are appropriate. Finally increased understanding of mechanisms of VEGF inhibitor resistance, including enhanced invasion and infiltration, is critically needed to further improve the outcome with anti-angiogenic therapy for malignant glioma patients.

## Figures and Tables

**Table 1:** Patient characteristics

**Tumor grade**	**3 (*n*=32)**	**4 (*n*=27)**
Age, median (years)	45.9	54.3
Range (years)	24.5–61.7	24.1–70.4
Gender, male	19 (59%)	17 (63)
Female	13 (41%)	10 (37)
		
*KPS*		
90–100	25 (78%)	19 (70)
70–80	7 (22%)	8 (30)
		
*Diagnosis*		
GBM		27 (100)
AA	18 (56%)	
AO	13 (41%)	
PXA	1 (3%)	
Time from diagnosis, median (weeks)	189.0	80.4
Range	11.3–1173.9	24–450
		
*No. of previous PDs*		
1	14 (44%)	14 (52)
2	9 (28%)	8 (30)
3	9 (28%)	5 (19)
Previous XRT	32 (100%)	27 (100)
		
*Surgery before enrollment*		
GTR	3 (9%)	1 (4)
STR	2 (6%)	0
Biopsy	2 (6%)	1 (4)
None	25 (78%)	25 (92)
		
*No. of previous ChemoRx agents*		
1	10 (31%)	6 (22)
2	8 (25%)	8 (30)
3	7 (22%)	6 (22)
⩾4	7 (22%)	7 (26)

Abbreviations: AA=anaplastic astrocytoma; AO=anaplastic oligodendroglioma; GBM=glioblastoma; GTR=gross total resection; KPS=Karnofsky performance status; PD=progressive disease; PXA=pilocytic xanthroastrocytoma; STR=subtotal resection; XRT=radiotherapy.

The numbers in parentheses refer to percentage unless otherwise indicated.

**Table 2:** Frequency of adverse events observed in ⩾10% of patients

	**Grade**			
**Toxicity**	**2**	**3**	**4**	**5**
Anaemia	11 (19%)	1 (2%)		
Anoraexia	6 (10%)			
Diarrhoea	6 (10%)			
Fatigue	18 (31%)	1 (2%)		
Hypertension	6 (10%)	1 (2%)	1 (2%)	
Infection	13 (22%)	4 (7%)	1 (2%)	
Mucositis	11 (19%)			
Nausea/emesis	11 (19%)			
Neutropaenia	15 (25%)	9 (15%)	5 (8%)	
Proteinuria	6 (10%)	1 (2%)		
Rash	6 (10%)			
Thrombosis		4 (7%)	2 (3%)	1 (2%)
Transaminase elevation	6 (10%)	1 (2%)		

**Table 3:** A comparison of outcomes of this study with other prospective clinical trials

**Number of patients**	**RR (%)**	**Median PFS (weeks)**	**PFS-6 (%)**	**Median OS (weeks)**	**Reference**	**Regimen**
*Grade 3*						
32	24	24 (16–33)	41 (24,57)	63.1 (36–∞)	Current study	BV + etoposide
33	61	30 (21–60)	55 (36–70)	65 (32–99)	Desjardins *et al* (2008)	BV + CPT-11
						
*GBM*						
27	23	18 (13–40)	44.4 (26–62)	46.4 (25–70)	Current study	BV + etoposide
35	57	24 (18–36)	46 (32–66)	42 (35–60)	Vredenburgh *et al* (2007a, 2007b)	7
85	28	17 (12–23)	43 (30–56)	36.8 (33–43)	Friedman *et al* (2009)	BV monotherapy
82	38	22 (18–25)	50 (37–64)	34.8 (31–44)	Friedman *et al* (2009)	BV + CPT-11
48	35	16 (12–26)	29 (18–48)	31 (21–54)	Kreisl *et al* (2009)	BV monotherapy

Abbreviations: BV= bevacizumab; GBM=glioblastoma; OS=overall survival; PFS=progression-free survival; PFS-6=progression-free survival at 6 months.

Numbers in parentheses refer to available 95% confidence intervals.
