# Supplementary figures and images for: Metronomic chemotherapy with daily, oral etoposide plus bevacizumab for recurrent malignant glioma: a phase II study
Source: Br J Cancer. 2009 Nov 17;101(12):1986–94. doi: 10.1038/sj.bjc.6605412 (PMC2795427; doi:10.1038/sj.bjc.6605412)

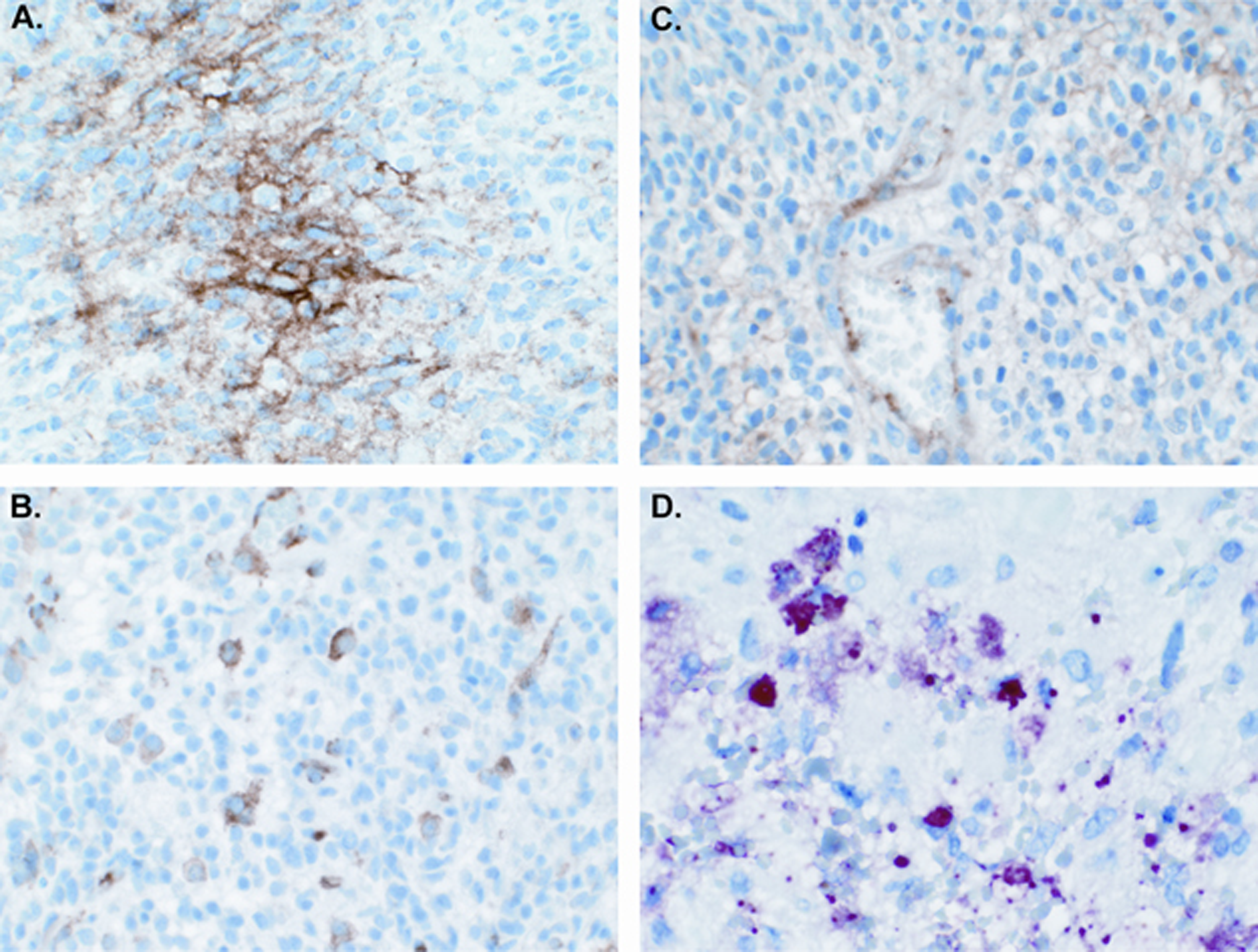

Supplement: Supplementary Figure [file 6605412x1.tif]
