# Supplementary material for: Metronomic chemotherapy with daily, oral etoposide plus bevacizumab for recurrent malignant glioma: a phase II study
Source: Br J Cancer. 2009 Nov 17;101(12):1986–94. doi: 10.1038/sj.bjc.6605412 (PMC2795427; doi:10.1038/sj.bjc.6605412)
Supplement: Supplementary Figure Legend [file 6605412x2.doc]

*Supplemental Figure 1.* Representative immunohistochemistry staining from archival tumor material for the angiogenesis markers vascular endothelial growth factor (VEGF) (panel A) and vascular endothelial growth factor receptor -2 (VEGFR-2) (panel B), as well as the hypoxia markers carbonic anhydrase-9 (CA9) (panel C) and hypoxia-inducible factor-2α (HIF-2α) (panel D) expression.
