# Supplementary material for: Metronomic chemotherapy with daily, oral etoposide plus bevacizumab for recurrent malignant glioma: a phase II study
Source: Br J Cancer. 2009 Nov 17;101(12):1986–94. doi: 10.1038/sj.bjc.6605412 (PMC2795427; doi:10.1038/sj.bjc.6605412)
Supplement: Supplementary Table [file 6605412x3.doc]

**Supplemental Table 1.** Tumor Biomarkers and Progression-Free Survival

| Biomarker | Histology | Cutoff | Number of Patients | 6-PFS (%) | 95% CI | p-value |
| --- | --- | --- | --- | --- | --- | --- |
| CA9 | GBM | ≤ 10% | 4 | 75.0 | 12.8, 96.1 | 0.037 |
|  |  | > 10% | 8 | 37.5 | 8.7, 67.4 |  |
|  | Grade 3 | ≤ 10% | 7 | 42.9 | 9.8, 73.4 | 0.965 |
|  |  | > 10% | 4 | 28.6 | 4.1, 61.2 |  |
|  |  |  |  |  |  |  |
| HIF-2α | GBM | ≤ 10% | 4 | 75 | 12.8, 96.1 | 0.970 |
|  |  | > 10% | 4 | 50.0 | 5.8, 84.5 |  |
|  | Grade 3 | ≤ 5% | 4 | 33.3 | 4.6, 67.6 | 0.464 |
|  |  | > 5% | 4 | 50.0 | 5.8, 84.5 |  |
|  |  |  |  |  |  |  |
| VEGFR2 | GBM | ≤ 20% | 4 | 50.0 | 5.8, 84.5 | 0.9 |
|  |  | > 20% | 8 | 50.0 | 15.2, 77.5 |  |
|  | Grade 3 | ≤ 10% | 6 | 16.7 | 0.8, 51.7 | 0.131 |
|  |  | > 10% | 5 | 80.0 | 0.4, 96.9 |  |
|  |  |  |  |  |  |  |
| VEGF | GBM | ≤ 30% | 4 | 0 | - | 0.006 |
|  |  | > 30% | 8 | 75 | 31.5, 93.1 |  |
|  | Grade 3 | ≤ 30% | 6 | 33.3 | 4.6, 67.6 | 0.185 |
|  |  | > 30% | 5 | 60.0 | 12.6, 88.2 |  |

Abbreviations: 6-PFS, progression-free survival at 6 months; CA9 – carbonic anhydrase nine; CI: confidence interval; HIF-2α: hypoxia-inducible factor two alpha; VEGFR2: vascular endothelial growth factor receptor 2; VEGF: vascular endothelial growth factor
